# Adaptation and qualitative evaluation of the BETTER intervention for chronic disease prevention and screening by public health nurses in low income neighbourhoods: views of community residents

**DOI:** 10.1186/s12913-024-10853-z

**Published:** 2024-04-04

**Authors:** Mary Ann O’Brien, Aisha Lofters, Becky Wall, Regina Elliott, Tutsirai Makuwaza, Mary-Anne Pietrusiak, Eva Grunfeld, Bernadette Riordan, Cathie Snider, Andrew D. Pinto, Donna Manca, Nicolette Sopcak, Sylvie D. Cornacchi, Joanne Huizinga, Kawsika Sivayoganathan, Peter D. Donnelly, Peter Selby, Robert Kyle, Linda Rabeneck, Nancy N. Baxter, Jill Tinmouth, Lawrence Paszat

**Affiliations:** 1 Department of Family and Community Medicine, Temerty Faculty of Medicine, University of Toronto, Fifth Floor, 500 University Ave, Toronto, ON M5G 1V7 Canada; 2 Women’s College Research Institute, Women’s College Hospital, 76 Grenville St, Toronto, ON M5S 1B2 Canada; 3 Peter Gilgan Centre for Women’s Cancers, Women’s College Hospital, 76 Grenville St, Toronto, ON M5S 1B2 Canada; 4 Dalla Lana School of Public Health, University of Toronto, 155 College Street, Suite 424, Toronto, ON M5T 3M6 Canada; 5 Durham Region Health Department, Regional Municipality of Durham, 605 Rossland Road East, Whitby, ON L1N 6A3 Canada; 6 Ontario Institute for Cancer Research, 661 University Ave, Suite 510, Toronto, ON M5G 0A3 Canada; 7 MAP Centre for Urban Health Solutions, St. Michael’s Hospital, 30 Bond Street, Toronto, ON M5B 1W8 Canada; 8 Department of Family and Community Medicine, St. Michael’s Hospital, 61 Queen St E #3, Toronto, ON M5C 2T2 Canada; 9 Department of Family Medicine, Faculty of Medicine & Dentistry, University of Alberta, 6 - 10 University Terrace, Edmonton, AB T6G 2T4 Canada; 10 Department of Pediatrics, McMaster University, 1280 Main St West, Hamilton, ON L8S 4K1 Canada; 11 School of Medicine, University of St Andrews, St Andrews, Fife, KY16 9TF UK; 12 Centre for Addiction and Mental Health, 1025 Queen Street West, 5Th Floor, Toronto, ON M6J 1H4 Canada; 13 Melbourne School of Population & Global Health, University of Melbourne, 207 Bouverie Street, Melbourne, VIC 3053 Australia; 14 Sunnybrook Research Institute, Sunnybrook Health Sciences Centre, 2075 Bayview Avenue, Toronto, ON M4N 3M5 Canada

**Keywords:** MeSH terms, Chronic disease, Primary prevention, Nurses, Public health, Qualitative evaluation, Social determinants of health

## Abstract

**Background:**

The BETTER intervention is an effective comprehensive evidence-based program for chronic disease prevention and screening (CDPS) delivered by trained prevention practitioners (PPs), a new role in primary care. An adapted program, BETTER HEALTH, delivered by public health nurses as PPs for community residents in low income neighbourhoods, was recently shown to be effective in improving CDPS actions. To obtain a nuanced understanding about the CDPS needs of community residents and how the BETTER HEALTH intervention was perceived by residents, we studied how the intervention was adapted to a public health setting then conducted a post-visit qualitative evaluation by community residents through focus groups and interviews.

**Methods:**

We first used the ADAPT-ITT model to adapt BETTER for a public health setting in Ontario, Canada. For the post-PP visit qualitative evaluation, we asked community residents who had received a PP visit, about steps they had taken to improve their physical and mental health and the BETTER HEALTH intervention. For both phases, we conducted focus groups and interviews; transcripts were analyzed using the constant comparative method.

**Results:**

Thirty-eight community residents participated in either adaptation (*n* = 14, 64% female; average age 54 y) or evaluation (*n* = 24, 83% female; average age 60 y) phases. In both adaptation and evaluation, residents described significant challenges including poverty, social isolation, and daily stress, making chronic disease prevention a lower priority. Adaptation results indicated that residents valued learning about CDPS and would attend a confidential visit with a public health nurse who was viewed as trustworthy. Despite challenges, many recipients of BETTER HEALTH perceived they had achieved at least one personal CDPS goal post PP visit. Residents described key relational aspects of the visit including feeling valued, listened to and being understood by the PP. The PPs also provided practical suggestions to overcome barriers to meeting prevention goals.

**Conclusions:**

Residents living in low income neighbourhoods faced daily stress that reduced their capacity to make preventive lifestyle changes. Key adapted features of BETTER HEALTH such as public health nurses as PPs were highly supported by residents. The intervention was perceived valuable for the community by providing access to disease prevention.

**Trial registration:**

#NCT03052959, 10/02/2017.

**Supplementary Information:**

The online version contains supplementary material available at 10.1186/s12913-024-10853-z.

## Background

Screening rates for cancers and other chronic diseases are suboptimal in Ontario, Canada [1, 2]. Moreover, studies show higher rates of chronic disease and lower rates of chronic disease prevention and screening (CDPS) activities in low income areas in Canada [3, 4]. For example, increased smoking and exposure to second-hand smoke are associated with lower income [4].

Canadians living with low income are more likely to develop chronic diseases compared to those with higher income. For example, Roberts et al. found that among Canadians in the 35–49 year age group, people in the lowest versus highest income quintile had an adjusted odds ratio of 7.5 [95% CI: 4.0–13.7] for multi-morbidity [5]. For people in the 50–64 year age group, the adjusted odds ratio for multi-morbidity was 5.9 [(95% CI: 4.4–7.9] in the lowest versus highest income quintile [5].

There is some evidence that specific interventions may reduce barriers to accessing preventive services in disadvantaged populations. For example, in a systematic evidence review aimed at achieving health equity in ten preventive services Nelson et al. found that patient navigation improved screening rates for breast, cervical, and colorectal cancers [6]. Other effective interventions for specific cancer screening included telephone calls and point-of-care prompts (colorectal cancer) as well as reminders from lay health workers (breast cancer) [6].

In Canada, the Building on Existing Tools to Improve Chronic Disease Prevention and Screening (BETTER) intervention has been shown to increase the uptake of CDPS activities in primary care in urban [7–10] as well as in rural and remote settings [11]. Briefly, the original BETTER intervention consisted of a one-time 1:1 visit between a specially-trained prevention practitioner (PP) and a patient (40–65 years). During the visit, the PP and patient reviewed recommended CDPS activities and through principles of brief action planning and shared decision-making, the PP assisted the patient to identify one to three personal goals [7–10].

Although the BETTER intervention has been shown to be effective, it has been conducted in primary care settings with full access to electronic medical records, in which study participants were already connected to a family physician. Moreover, in the original BETTER trial, about half of the participants had an income of $100,000 (CAD) or higher [7]. Since a large number of Canadians do not have access to a primary care practitioner [12] and it was unknown if the BETTER intervention would be effective for people living with low income, we adapted the BETTER intervention to a public health setting (without access to electronic or paper medical records from any source) with public health nurses as PPs, and conducted a cluster randomized controlled trial (cRCT) that compared the adapted BETTER intervention to a wait-list control [13, 14]. We previously reported that six months after the prevention visit participants in the intervention arm met 64.5% of actions for which they were eligible versus 42.1% in the wait-list arm (rate ratio 1.53 [95% confidence interval 1.22–1.84]) [14]. In that cRCT, more than 90% of participants had an annual household income of less than $60,000 (CAD) [14].

This paper describes two study phases, 1) the process for adapting the intervention from primary care to a public health setting; and 2) the post-visit qualitative evaluation by community residents. We refer to the adapted intervention as ‘BETTER HEALTH’, the first implementation of the BETTER intervention outside of a primary care setting. Details of the adaptation process could be useful to others who are interested in implementing the BETTER HEALTH intervention in their own setting for individuals who may not have access to a primary care practitioner. We also conducted a qualitative evaluation of the intervention by community residents. We undertook this evaluation to complement the results of the cRCT to understand how residents viewed the intervention and how they made lifestyle changes to reduce their risk of chronic disease. Widespread implementation of the BETTER HEALTH intervention in a public health setting may contribute to a reduction in health inequities by facilitating access to prevention services and lifestyle advice.

## Methods

### Setting

Durham Region is located within the eastern portion of the Greater Toronto Area in Ontario and comprises eight municipalities with an estimated population (2018) of 683,600 [15]. Population health assessments by the Durham Region Health Department (DRHD) showed high rates of chronic disease and smoking, and low cancer screening rates in seven neighbourhoods with low-income levels, deemed as priority health neighbourhoods [16]. For example, the health neighbourhood of Downtown Oshawa had a breast cancer screening rate of 55.3, cervical cancer screening rate of 52.5 and rate of overdue for colorectal cancer screening of 58.1 (2016 age-standardized rates per 100) [16]. By comparison, Ontario age-standardized rates for breast cancer screening, cervical cancer screening, and overdue of colorectal cancer screening were 64.5, 62.0, and 38.1 respectively [17].

The public health setting was an appropriate fit for the adapted intervention since chronic disease prevention and well-being are part of the Ontario Public Health program standards [18].

### Approach

In preparation for adaptation of BETTER from primary care to public health, principles of community-based participatory research (CBPR) were used to design a community engagement strategy [19, 20]. Key elements included close collaboration with public health partners to identify a range of community stakeholders, creating the study Community Advisory Committee (CAC) (*n* = 14) that included representation from public health (*n* = 5), service agencies/social services (*n* = 4), primary care (*n* = 2), and residents of low income neighbourhoods (*n* = 3). We also received advice from a Primary Care Engagement Group (*n* = 9) that included family physicians (FPs), a nurse practitioner (NP), and public health staff to provide advice on the adaptation of BETTER, recruitment strategies and approaches to community engagement. The CAC was engaged throughout the study period and met in-person approximately three times per year. They provided advice on all aspects of the study design especially for recruitment in low-income neighbourhoods and fit with existing community services. For example, the community resident members of the CAC reinforced the importance of being treated with respect, the value of recruitment in public spaces such as libraries, and provided suggestions for helping with referrals (if desired by participants). Other examples of CAC involvement included connections to not-for-profit housing and access to a food bank for recruitment. The Primary Care Engagement Group helped identify local family physicians or a nurse practitioner willing to accept new referrals for study participants (if desired). They also helped connect the study to other primary care practitioners in the region [13].

In both study phases, we used purposeful sampling [21, 22]. This approach is appropriate in a qualitative research study when the purpose is to obtain information from participants who are knowledgeable about the topic under investigation [21, 22].

### Adaptation phase

We used the ADAPT-ITT model (Assessment, Decision, Administration, Production, Topical Experts – Integration, Training, Testing) for the adaptation [23]. Table 1 summarizes how the steps were applied. Briefly, the research team conducted an initial assessment by reviewing the recruitment strategies and components of the PP visit and BETTER toolkit (educational materials, a ‘Prevention Prescription’, ‘Bubble Diagram’ and “Goals Sheet”), and then made preliminary adaptations. For example, we revised the BETTER toolkit to include community resources such as support for income and food insecurity. Public health nurses were identified as PPs instead of practitioners from within primary care practices. Next, eligible community residents reviewed the adapted intervention during focus groups and interviews and provided feedback, as did the CAC. We incorporated community resident and CAC recommendations and further refined recruitment strategies such as displays at local community events. The PP training was based on the adapted features. Key adaptations included recruitment via numerous community facilities and events rather than by primary care practices; baseline data collection by self-report and collected by a supportive research assistant during an interview; participants in both arms received the standard educational materials from the DRHD; the prevention meeting and interaction with the PP was adapted to include a 'warm hand off' referrals for CDPS; and the location of the baseline and outcome assessment and the prevention meeting were all at the venue chosen by the resident.

**Table 1:** Description of steps in ADAPT-ITT^a^ framework for adaptation of BETTER^b^ from primary care to a public health context

Step in ADAPT-ITT	Application to BETTER HEALTH	Adaptations
Assessment	Initial assessment by the study team based on expertise and using the results of an existing structured literature review: - reviewed the BETTER participant recruitment strategies - reviewed the baseline survey content and delivery method - reviewed Prevention Practitioner (PP) visit & toolkit vis a vis the study population	Irrelevant questions removed from baseline survey Survey further adapted to include items about living in poverty, food insecurity, and social support Baseline survey administered in person by research assistant Public health nurses designated as PPs PP toolkit revised: - included community resources for social services support e.g. for income and food insecurity - minor revisions to Prevention Prescription (a take-home document for participants written by PPs summarizing reminders and referrals for CDPS activities), including asking about social determinants of health Changed visit location from physicians’ offices to a place preferred by community residents and PPs e.g. local libraries and residents’ homes if they chose
Decision	Decision by research team to proceed to administration step based upon review of initial adaptation	
Administration	Interviews and focus groups with target population to understand barriers and facilitators to disease prevention and lifestyle modification and explore elements for adaptation to BETTER HEALTH	Qualitative results from community residents: Supported public health nurse as PPs who were viewed as knowledgeable and able to provide information and linkages to community services and/or primary care Reinforced changes to PP toolkit i.e., include community resources for social services (e.g., income support) and other resources (e.g., shelters, community kitchens, mental health supports) –and help with referrals (if desired by participant) PP visits to be confidential and 1:1 Location of visit: space mutually agreed upon by resident and PP
Production	Production of a further refined version	N/A
Topical experts	Study team; key stakeholders in Durham Region, Ontario; public health Prevention Practitioners, and community residents living in low income neighbourhoods (CAC), further refined the recruitment strategies for the trial	Incorporated study displays at local community events Retained intervention components that had been adapted by the research team
Integration	A final adapted version that integrates all findings	Adapted intervention was used in the trial
Training	Training of current PPs on adapted BETTER HEALTH	N/A
Testing	Qualitative evaluation by community residents post visit; quantitative testing in cluster randomized controlled trial	Due to time constraints, we did not pilot test the adapted intervention

^a^ ADAPT-ITT model: Assessment, Decision, Administration, Production, Topical Experts – Integration, Training, Testing

^b^ BETTER: Building on Existing Tools to Improve Chronic Disease Prevention and Screening

#### Inclusion criteria

Community residents 40 to 64 years old were eligible for inclusion in adaptation focus groups and interviews if they lived in identified priority health neighbourhoods and were English speaking. We chose the age range of 40 to 64 years for the adaptation so that we would obtain views from people who were in the same age range as those who would be eligible to receive the PP visits. We reasoned that it was preferable to make any adaptations to the visits or program materials if recommended by people in the same age range rather than by those younger or older who might not be eligible for a given screening test.

#### Recruitment

Residents were recruited through flyers and posters distributed at libraries, community drop-in centers, community kitchens, community events, libraries, and shelters. Recruitment also occurred at in-person presentations in the community, via advertisements in local newspapers, and by word of mouth.

### Post-visit qualitative evaluation phase

#### Inclusion criteria

Community residents were eligible to participate if they were part of the study intervention arm, and had completed the PP visit and 6-month data collection. Community residents who participated in the adaptation phase were not eligible for the visits or the post-visit qualitative evaluation.

#### Recruitment

Residents who had enrolled in cRCT intervention arm and agreed to be contacted for participation in the qualitative evaluation were approached by email and/or telephone.

##### Data collection

For both adaptation and post-visit qualitative evaluation phases, we conducted focus groups and interviews (adaptation, June to September 2017; evaluation, March to July 2019). We first recruited residents to focus groups, aiming for 4–8 members in each group [24]. When a resident wanted to participate but could not attend any of scheduled focus groups, we offered an individual interview instead. Interviews took place in a location chosen by the resident such as their home or a community space. All focus groups took place in a meeting room in a library or community centre that was accessible by public transportation. For each phase, we created a focus group and interview guides that were based on the study objectives then pre-tested by community residents living in low income areas who were members of the CAC. During all focus groups and interviews, we asked residents about their physical and mental health, impressions of their neighbourhoods, and their knowledge of and access to community resources and primary care. We also asked about the proposed visit structure, and appropriateness and completeness of the PP tools. For the post-visit evaluation, we asked residents about their views of the adapted BETTER HEALTH intervention including the administered survey and PP visit. All sessions were recorded, transcribed verbatim and anonymized. Sessions were conducted in-person by experienced qualitative researchers (MAO and TM) and lasted between 24 and 110 min. Community residents received a $25.00 (CAD) grocery gift card and two transit tickets in recognition of their time.

### Analysis

The adaptation and post-visit evaluation data were analyzed separately then combined. An inductive approach using the constant comparative method was used to analyze data [25, 26]. Initially, three team members (MAO, TM and SC) independently coded two transcripts, then met to compare coding, discuss differences and develop consensus on codes. Subsequently, two team members coded the remaining transcripts using the coding guide. We compared initial codes to each other within the same transcript and across transcripts in the adaptation phase and then in the post visit evaluation. As we developed the emerging themes from the coded data, we compared themes within a transcript then across transcripts looking for supporting as well as disconfirming instances. Team members met periodically with co-investigators DPM and NS to review and refine the coding manual, interpret findings, develop emerging themes and ensure consistency. NVivo 10 (QSR International) software was used for data management. An audit trail was used to ensure transparency of major analytic decisions [27].

We provide additional details about the qualitative methods in the ‘Consolidated criteria for reporting qualitative studies’ (COREQ) checklist [28]. (online Supplemental File 1).

#### Ethics

This study was approved by the Research Ethics Boards of the University of Toronto (# 33340), Sunnybrook Health Sciences Centre (REB 222—2016), St. Michael’s Hospital (REB #16–231) and Ethics Review Committee of the DRHD (ERC #20160802–002). Written informed consent was provided by all community residents prior to their interview or focus group. We also provide sample interview and focus group guides online in Supplemental File 2.

## Results

A summary of the key features of BETTER that were adapted for the BETTER HEALTH cRCT is provided in Table 1.

### Adaptation and post-visit focus groups and interviews

During adaptation, 4 focus groups and 5 in-person interviews were conducted over four months (14 community residents, 64% female; average age 54 y [range: 42 – 62 y]). For the post-visit qualitative evaluation, 6 focus groups and 2 in-person interviews were held over five months (24 community residents, 83% female; average age 60 y [range: 43–63 y]). On average, the focus groups and interviews were held 10 months after the visit. Three participants withdrew in the adaptation phase: one participant dropped out after a focus group because they decided it was not useful to them. Two potential participants declined to proceed with a focus group prior to its start because they did not wish to be identified on the consent form. No participants withdrew from the post-visit qualitative evaluation. All participants lived in one of the priority health neighbourhoods in the town of Whitby or city of Oshawa, ON, Canada.

### Major themes

We integrated the adaptation and post-visit results since community resident views of their health challenges were similar. We identified five themes and associated subthemes. The major themes were: 1) Significant intersecting health and social challenges in coping with everyday life; 2) Personal desire to change and readiness for change were key to improving health behaviours; 3) Value of accessible community programs and resources; 4) PPs enabled residents to change health behaviours through a client-centred education and goal setting approaches; and 5) Feeling listened to and being understood were critically important when interacting with PPs. See Table 2 for exemplar supporting quotes for each theme. We provide additional illustrative quotes in the sections below.

**Table 2:** Themes and Exemplar Quotations – Adaptation and Post- Visit Phases of BETTER HEALTH

Themes and Sub Themes	Adaptation Phase	Post-Visit Evaluation Phase
**Theme:** **1. Significant intersecting health and social challenges in coping with everyday life**		
a) Living in poverty	*[Interviewer asked about the health of people in the neighbourhood]* *Participant: ****“****Generally, it’s poor. There’s a lot of people running around broke, myself included. I get my cheque, I pay my rent, I pay my phone, and I get my bus pass.”* (Focus Group (FG) 2)	*[Interviewer asked if people in the neighbourhood were able to make ends meet]* *Participant: “You’re always trying to figure out how you’re going to get your food, how you’re going to get to the food bank. You know, what food bank…like what time of the month is it? Am I allowed to go again? Like it’s just your whole life is on survival. You can’t look past it.”* (Interview 1)
b) Coping with stressful lives	*[Interviewer asked about the health of people in the neighbourhood]* *Participant: “Well, there’s probably lots of health problems. Probably a lot of drug abuse. There's probably a lot of nutritional problems…I think they're just too sick. It’s they’ve got a sickness, a disease. They just… They can't fight it. Physically and mentally, emotionally maybe…And we’ve got a big homeless problem here.”* [Interviewer] *“So I wonder if you think there may be a connection there in terms of like people living on the streets and people abusing drugs or people not eating well.”* [Participant] *“Yeah, it’s all connected* [addictions, poverty, poor nutrition]*…yeah for sure it is. People just give up.”* (FG4)	*[Interviewer asked about the impact that stress has on wellbeing]* *Participant: “I suffered* [job] *burnout like a couple of years back. And it was devastating, right. Like I mean it literally threw me to the ground…And you think that, oh, you’re fine. And then you come home and that’s it, like then you realize all that stuff that you were taking in and you weren’t doing proper self-care.”* (FG5)
c) Being socially isolated & experiencing loneliness	*[Interviewer asked about the health of people in their neighbourhood. Participant 1 discussed poverty, then Participant 2 talked about sadness and loneliness]* [Participant 1]”*I’ve got one* [homeless person] *living with me right now. I’m putting him up because he was sleeping in an abandoned house. And I met him in the hostel.”* [Participant 2]. *They’re pretty poorly, aren’t they? Don’t you see? You see a lot of sadness, you see a lot of loneliness.”* (FG2)	*[Interviewer asked about people taking walks in neighbourhood. Participant commented that they see people more during Spring and Summer months. They went on to describe concerns about isolation]* *Participant: “I think that’s one of the real downfalls now of the way we’re all going. We’re all kind of locking ourselves up in our homes and we’re not getting out there… Because social isolation they say is just as bad as smoking, right, to your health.”* (FG2)
d) Living with mental health issues (perceived depression & anxiety)	*[Interviewer asked if the Participant thought they were at risk of developing chronic disease] Participant*: *“I’ve got depression, anxiety. Yeah, lots of those kind of things. “* (Interview 4)	*[Interviewer”:… what do you think causes some people to be healthy and some people unhealthy?”]* *Participant: “Well, I gained like, I don't know, 60, 70 pounds because I was stressed out. Really stressed out. So I went to chocolate. And I didn’t sleep. So it all works together, right. And even now like I lost 21 pounds and then in December, Christmastime, and then I kind of spiraled down because just my circumstances and my mindset against that, and lack of sleep and whatever. And I was trying to do too much, and I felt overwhelmed.”* (FG3)
e) Addictions	*[The Participant described what they viewed as ‘unhealthy’ behaviours in their neighbourhood]* *“They’re just partying in the streets because they don’t …That’s the way they know how to live, is daily drugs and they don't have no food. I don’t understand why they wouldn't buy food. But they have a disease, right?”* (Interview 1)	*[The Interviewer asked about unhealthy behaviours in the neighbourhood]* *Participant: “…I mean not just alcohol, we’re talking about, you know, drugs, pills, cocaine, whatever… And until they can face it* [addictions] *and try to get, you know, a grip at least on their addiction, try to do some harm reduction, it’s just you just… You know, I wouldn't eat for a week. You know what I mean? Like you can’t think straight and try to solve problems. So the first step was trying to get my harm reduction on my drug addiction. Trying to get a little control of it….”* (Interview 1)
**Subtheme: Disease prevention was a lower priority for many**	*[Interviewer asked about views on meeting with a nurse in the community to improve their health]* *Participant: “I think it’s* [disease prevention] *very important. I think it’s a great idea because people just… Some people are… What’s the word I’m looking for? Some people are very obsessed with certain parts of their life, and their health kind of just seems to take a backseat to everything.”* (Interview 5)	*[Interviewer asked about linkages between struggling to make ends meet and other health issues]* *Participant: “You know, when you’re in a position where things are fairly relaxed, you can look ahead because you’re not as concerned about the immediate. The immediate’s taken care of. And I think that that really places a lot of burden on their health in particular. And I know that among them, among people who are impoverished too, that smoking is more, drugs are more, more dangerous behaviours, less self-care, less ability to have self-care.”* (FG6)
**Subtheme: Different attitudes toward disease prevention in men compared to women**	[Participant 2]”*I’ve had my same doctor, honey, for the last 20 years. I can’t see myself going to any other doctor because…”* [Participant 1] *“It’s a guy thing too. Guys don’t go to a doctor as much or they wait longer to go.”* (FG2)	*“The thing is I always thought women are better at seeing doctors than men are. Men tend to avoid it at all cost…I think for men, a lot of times it’s admitting to somehow being weak because you’re ill. Which is stupid. I think women are a lot more intelligent about their health than men are. Men just ignore things. Like I mean how many times have you been sick and you just to hell with it, you know, I’m not even taking the day off. I’m going into work and I’m just going to persevere through it.”* (FG1 (male))
**Subtheme:Social influences on health—the “company you keep”**	*[Interviewer asked for feedback on BETTER toolkit. The participants described ways to improve health]* *Participant: “I talked to these people that are involved in this [walking group], and they developed relationships and what have you around these groups. And they’re doing activities… And you’d walk 16 k [kilometres] or 10 k or 5 k or 10 k. What you want to do is you want to form groups that are social…”* (FG3)	*Participant 1: “Yeah, I used to have 50 or 60 [friends]. Now I’ve got 5 because I don’t want to party and drink and smoke anymore, right. So it is, it’s a social thing.”*[*Participant 5: “A question, is it good to be healthy and not having friends?”* [laughs] *Participant 1: “Well, I’m not going to preach but I have a church that is very… Yeah, I find the community really good there.”* (FG2)
**Theme:** **2. Personal desire to change (i.e. being motivated) and readiness for change were key to improving health behaviours**	*[Interviewer asked about making choices to live healthy lives as compared to unhealthy lives]* *Participant: “I think that you have to kind of get to a mental state to be happy, and then you know that you can fix your body. You have to know in yourself that what is bad is not bad forever. It's only how you feel. And then you make that choice that I want to feel better.”* (FG2) [Interviewer] *“I think you’re a very good example in that you do take those steps to look after yourself. What makes you so different from other people though?”* [Participant] *“They (others) just don't have…you know, the get up to say, you know what, I want to change, you know.”* (FG4)	*[Interviewer asked about advice on how to follow through on personal preventive care goals]* *Participant: “For me it was just an understanding that my health was not good and I needed to change. I think an acceptance that, you know, you’re not perfect and that you can improve and you have to stay focused on it. You have to want it… And to me I just had to understand that it was not a good position I was in and it had to change. It's desire to change, I think.”* (FG1)
a) Residents found it difficult to change health behaviours	*[Interviewer asked if there was anything that participants had tried to do to improve their health]* *Participant: “Biking is good. It’s something that I don’t do but I should do all these things. I know what I should do. I just don’t do it. But I know what I have to do. I just don’t do it. Laziness… I’m lazy. I’ve got to get off the couch. I’ve got to put the remote down and start doing things, you know. I know I have to get healthy. It’s very difficult. Yeah, it’s very difficult.”* (FG2)	*[Interviewer asked if people were worried about developing chronic disease such as high blood pressure]* *Participant: “And I have had breast cancer. And I worry… I don’t… Like I take my blood pressure on a regular basis. There are things that I should be doing that would make that better. In terms of activity, it would make it better than it is. And I don't know why I’m not. Because I know. I know it logically.”* (FG6)
b) Wanting better health as motivation	*[Interviewer asked about motivation to make lifestyle changes]* *Participant: “I want better health. I’m getting older and you feel ouches and ouches with the weight. It’s not so much… It's not to be…to lose weight and look, you know, sexy or anything, it’s for my heart, for my organs and stuff, I want to eat healthy, so I can live longer.”* (Interview 1)	[*Interviewer: “So in your opinion what leads some people to be healthy and some people not to be healthy?]* *Participant 2*: *“I think in my opinion it’s people that want to prolong their life, that don't want to be hospitalized or, you know, on medication. I think that’s my fear. That’s why like I’m trying to take a step. Because as I said, I don't want to be on medication. Or like where I see my friends that are on… “Oh, I’m on this and this and this and this.” And I’m like, wow, like you know, you’re younger than me and you’re on like medication that…so many different types of medication. But I’m trying to eat healthy with a small budget.”* (Interview 2)
c) Readiness for change was identified as an important factor in changing health behaviours	*[Interviewer asked about making choices to live healthy lives as compared to unhealthy lives]* *Participant: “I think that you have to kind of get to a mental state to be happy, and then you know that you can fix your body. You know, I’ve been through so many ups and downs…And then you make that choice that I want to feel better.”* (FG2)	*“I worry from the smoking. Now, if somebody said I got cancer tomorrow, would I quit smoking? I always thought I would. But you know, I don't know. Like what does it take? It’s just one of those things that the heart and the head haven’t gotten together.”* (Interview 1)
d) The right timing was a key contributor – participants became aware of BETTER HEALTH at a pivotal time in their lives when they were primed for change	*N/A*	*“ I think for me, it was really good timing. As I said, I had just retired. So I was thinking, well, how are things…what am I going to do in the next… You know, how will I keep myself busy and occupied? So it was really good timing, that I was quite motivated to look at some…look at where my life was and what I wanted.* (FG4)
e) Participants perceived that a “wake-up call” or health scare provided motivation to change health behaviours; otherwise disease prevention was not a high priority	*Participant 1*: “*I quit drinking, six months ago maybe?” Participant 2*: *“Because he almost died. He was in the hospital. He had to go to the hospital.”*[*Participant 1:* “*My liver and everything shut down.” [Interviewer: “So that was the wake-up call?”] Participant 1*:*“Yeah.”* [laughs] (FG4)	*“A few years ago I had a stroke on April Fool’s Day. It was a perfect day for… I love that day. It was a beautiful day. But my blood pressure was high but I didn’t bother doing anything about it. It was a big mistake. I didn’t do a thing. And I found out the hard way. A blood clot in the brain. It messes you up. That’s what the education was in the hospital. I didn’t know salt was the cause, and smoking and caffeine. I knew that. What can I say, live and learn.”* (FG4)
**Theme:** **3. Value of accessible community programs and resources**	*Participant: “Now that I’m financially tied [limited finances], I find I go to the library a lot more. I don’t take books out as much but I go there for all my movies, right. I go there to read the newspapers. And you know what, a lot of people who are on fixed incomes or aren’t working or whatever, that’s where they go too. They go there to get on the computers, they go there to see you*.(FG1)	*“As I was preparing for retirement, I found that very distracting and stressful – about what that was going to look like and I’d be very lonesome. So I joined the seniors centre before I retired. Well, I’ve done stuff with them – with the seniors centre…”*(FG4)
**Subtheme: Valuing guidance and assistance to connect to resources**	*Interviewer asked about meeting with someone to help make different lifestyle choices or referrals}* *Participant: “…Like I got lucky. Somebody actually told me, ‘did you know?’ I had somebody take me by the hand, this guy, this big guy, the nicest guy you could ever ask, ‘this is where you go to eat’. Because I didn’t know. I had lived as a shut-in for 5 years.’This is where you go.’ He took me down to St. Vincent’s. And I went, ‘Oh wow, really! This is where you go for lunch’. Make people aware.”* ( FG2)	[Interviewer: *“So did you ask for resources within the community that would have maybe aligned with what you were more interested in?]* *Participant: Yes. And she (PP) engaged me at that level. And also returned some telephone calls when I asked about, for instance, salt water pools – are there any salt water pools here in Durham? … Or eating seasonally and eating organic vegetables as much as possible, and how difficult that is. And part of that being that it’s hard for me to get a farmers market. Or even for a CSA, a farm share – Community Shared Agriculture – it's hard for me to go to the farm and pick that up on a weekly basis or a bi-weekly basis or whatever. And I said nobody delivers. You know, they deliver to downtown. And she said, “No, [name of company] Organics delivers.” So great, so she put me in touch with [name of company] Organics.”* (FG3)
**Theme:** **4. PPs enabled participants to change health behaviours through a client-centred approach to education and goal setting**		
a) Reported health behaviour changes i. Participants described making positive changes including more exercise, quitting smoking, more social connections	*N/A*	*Participant 1: “I started doing exercises* [After PP visit].” *Participant 2: “I’ve got guinea pigs. So I said that’s a good way for me to start eating vegetables. Because they eat the vegetables so I have to constantly buy and stock up on vegetables. So I said I’ll eat with them* [laughs]. *I’ve got to get some willpower.”* (FG1)
ii. The majority of participants had *immediate* follow through on some goals but not everyone still maintained changes after 1 year	*N/A*	*“I actually did go to my doctor after the encouragement by the nurse. I actually went… I did it more frequently. And I made some tests like in advance because of…it was suggested by the nurse.”* (FG1)
b) The PP enabled participants to make *changes* i. Participants appreciated the process of setting small goals which were tailored to them	*N/A*	*Participant 1*:…*like I would say, oh, I want to lose like maybe 10 pounds over 6 months. And then she [Nurse] made me break it down to like okay, you lose .5 pounds every week or something like that. [laughs] Participant 2*: *Yeah, she did that with me too.*[*Participant 1: She’d get you to be real. [laughs]* *Participant 2: Yeah”* (Participants, FG5)
ii. The tools used by PPs were perceived as accessible, easy to use and provided good reminders for participants	*“I think it’s* [prevention prescription] *terrific because… I don’t really know many people who know their blood pressure. I don't know my cholesterol. I don't know anybody who does know their cholesterol…But to have this, yeah. Like for a lot of people that I know, like if you could just put it down on a piece of paper and put it in front of them, like they might change. They’ll take notice. They will make an effort. Or not, you know. But if it’s there right in front of them, you know, they can sort of look at it as like, you know, tests don’t lie, facts are facts. It's in front of you and it’s yours.”* (FG1)	*“I only realized I hit my goals because I kept them like you [referring to another FG participant]. I kept them posted. I knew and understood what they were. And I brought everything, all those sheets, I brought them to my doctor. And he went through everything. And he kind of kept me focused too.”* (FG1)
iii. The PP visit was perceived to have value by educating participants (a type of “wake-up call”) about their health and encouraging positive health behaviours	*“If you don’t have the vision to want to be healthy, you’re not going to go that route. But I think if you get a little bit of a push, talking to people or assisting you, you start thinking, you know, let me eat healthier. You know what I mean?” (*Interview 1) *“Because some people need that push. Some people might listen to a professional instead of their friend. Because I do have a friend that is overweight. She needs to lose that weight. But there's nobody there… I mean I tell her. She’s not going to listen. Like some people are stubborn. But sometimes when it’s explained, they might listen.”* (FG3)	*“Well, I thought about it, right. About changes. And she* [public health nurse PP] *was the wake-up call. Like I knew I had to eat more vegetables. I knew I had to eat less fat. So that part, you know, I knew I had to make a change there. But hearing her say it, it seemed like it was more of a goal rather than when I was on before I saw her… So I know. I know what has to be done. But the little push came from her when she was explaining to me about the vegetables, incorporating all the vegetables with your protein, and stuff like that. She made it sound a lot easier because I’m not a cook.”* (FG5)
iv. Public health and public health nurses were trusted sources of health information	*N/A*	*“Just to have someone, like another woman [referring to public health nurse PP] who’s knowledgeable about what’s important and about health, and to be able to help me walk through some of the extra steps that I need to take. And to give me more information. Because I used to think that the mammogram was… Like by the time you have a mammogram, if they detect it, it’s too late, you know. And she said, “No, it’s quite the contrary.” So that was like a misconception on my part. And she did educate me.”* (FG3)
**Theme:** **5. Feeling listened to and being understood was critically important when interacting with Prevention Practitioners (PPs) about their health**	***“****Well, it helps me* [meeting one-on-one with nurse in the community] *because then I walk away…I feel like I’m more… I used to be shy at one point. But I’m only shy with some people now when I meet them at first. And then once I get to know them, I’m fine. But it’s just that initial reaction. Should I trust them, are they going to tell everybody my secrets?”* (Interview 3) *“I like that one-on-one interaction. I think it’s important that I can sit there … and tell her what my goals are and what my aspirations are.”* (FG1)	*“And like with the nurse, I remember saying… She says, “No, you can ask any question.” And like she was listening and she was taking her time. It’s not the same as being in a doctor’s office where it’s rush, rush, rush… So I think it’s very important for people to have like a rapport, like a more slower pace. I think it’s important. And then you can get more information and go at your own pace”* (FG3)
a) The PP was perceived as a health professional with knowledge and skills to provide disease prevention care with knowledge of community resources	*[Interviewer asked about views on potential visits with a PP public health nurse]* *Participant: “…having a professional who is informed about the system, the big picture. ‘Oh, did you know that you can get this done? Well, yeah, we’ll make that appointment for you.”* (FG2)	*[Interviewer asked about views on visit with PP public health nurse]* *Participant: "Like I think that if you had a regular… If you have a regular practitioner, I don't know that it has to be a doctor. And I think that that’s a good use of that. Because she [PP] had wonderful, wonderful knowledge.”* (FG6)

### Significant intersecting health and social challenges in coping with everyday life

Participants described five significant challenges that affected their health: a) living in poverty, b) coping with stressful lives including difficult work or social environments, c) being socially isolated and experiencing loneliness, d) living with depression and anxiety, and, e) living with addictions to alcohol or drugs. Residents described the effects of living in poverty such as not having enough money to buy nutritious food, for example fresh fruits and vegetables, and feeling stressed by having insufficient resources to make ends meet. They also perceived other intersecting influences in their lives such poor living conditions, mental illness and unemployment which could lead to drug or alcohol addiction and ignoring health problems when they occurred.

“There’s a lot of homeless in my area, mental health issues. People can’t fix themselves if you don’t have good medical around or money to go to it… Because if you don't have food and you don’t have money, you go into depression.” (Adaptation, Interview 1)

#### Subtheme: disease prevention was a lower priority

As a result of health and social challenges, residents described that disease prevention was a lower priority. They described that they were likely to wait until they became ill, rather than pre-emptively engage in disease prevention. Other residents said they had to be in a “good place” before they could take steps to improve their health.

*“And I really think that people don’t take preventive maintenance that readily… I really don’t think so. …not until they get it* [illness]….*that’s me personally.”* (Adaptation, Focus Group (FG) 3)

#### Subtheme: different attitudes toward disease prevention in men compared to women

Both men and women said that men were less likely than women to focus on disease prevention. Generally men did not want to admit to ill health which they perceived as a weakness. Men were also skeptical about the value of disease prevention and less likely than women to think that it should be a priority.

#### Subtheme: social influences on health—the “company you keep”

Throughout both phases, residents described how social connections influenced their health. Being engaged in the community, and finding purpose in life were associated with taking steps toward better health. Others described how their circle of friends had negative influences on health behaviours by encouraging smoking and alcohol habits. As a consequence of choosing healthier behaviours, some residents described that their circle of friends had diminished.

*“I was going to say – the company you keep, right? … Yeah, it makes a big difference. Right? Because if your friends are drinking, you will drink. If your friends are smoking, you may smoke. And even if you’re not smoking, you’re inhaling that smoke, right. So it makes a difference.”* (Post-Visit, FG5)

### Personal desire to change and readiness for change were key to improving health behaviours

During both phases, residents described the importance of motivation and readiness to change.

They described that: a) it is difficult to change behaviours, b) the desire for better health is a motivator, c) that readiness to change is an important factor in changing health behaviours, d) the timing when they were primed to change was important and e) that a “wake-up call” may provide motivation to change. Several residents described internal motivation as important in making changes — that one had to make a choice to change their behaviour. Lack of motivation was identified as one reason why people do not change; people may know what to do to improve their health, yet often do not modify their behaviour. At the same time, residents acknowledged that it is very difficult to change behaviours that contribute to poor health. For example,

*“I know I have to get healthy. …It’s very difficult. My obstacle is my big stomach. It’s hard to get motivated to get started.”* (Adaptation, FG2)

During both phases, residents reported on previous attempts to improve their health if they had experienced a health scare or what was often referred to as a “wake-up call” that motivated them to make changes. For instance, several residents became aware that their blood pressure was elevated or that they had gained more weight than they had expected. During adaptation, residents described having taken different strategies to improve health such as walking, biking, and using community gardens for fresh vegetables. Walking and biking activities were described as essential since most residents could not afford a car. In the post-visit phase, residents described that wanting better health for themselves was a significant motivator to join the study, and some had already started to make changes prior to the PP visit. For many, the right timing was identified a key contributor to motivation – participants became aware of BETTER HEALTH at a pivotal time in their lives when they were primed for change.

### Value of accessible community programs and resources

During adaptation, residents mentioned different community programs including food banks, community kitchens, libraries and community centres that provided much-needed resources (e.g. food and clothing) and referrals to service agencies such as John Howard Society (a non—profit organization focused on education and community service pertaining to criminal justice systems), Legal Aid, and the Canadian Mental Health Association. The perception was that educational programs and community resources helped people in the neighbourhood become healthier.

*“And yes, you can eat well.* [Name of city] *is very good for that if you put your mind to it and get into their time schedules. The churches once a month do a soup and sandwich right there on* [name of street] *right, like right across from the library.* (Adaptation FG3)

Importantly some residents did not know about community programs and many residents had difficulty obtaining relevant and accurate information about chronic disease prevention and health care outside of the PP visit.

#### Subtheme: valuing guidance and assistance to connect to resources

During adaptation, residents perceived that they needed someone to help them to navigate health care and social systems by assisting them to connect with health or social resources and getting appropriate referrals e.g., for help with mental health issues. Some residents described positive experiences of receiving help from both peers and professionals, and getting connected to local services.

*“I just found out I can see a psychologist to deal with my head issues for free as long as it’s a referral from* [name of clinic]. (Adaptation FG2)

### Prevention Practitioners (PPs) enabled residents to change health behaviours through a client-centred approach to education and goal setting

In both adaptation and post-visit phases, residents perceived the PP as a health professional with knowledge and skills to support disease prevention. In adaptation, residents also liked that PP visit would be private since confidentiality was important. Residents reported that: a) their health behaviour changed, and b) that the PP enabled them to make changes.

Residents in the post-visit phase described making positive lifestyle changes as a result of the PP visit such as exercising more often, quitting smoking, and making more social connections. The majority of participants said they had immediate follow through on some goals. Sustained follow through was mixed; some had not continued with their goals but wanted to get back on track while others had continued to maintain behaviour changes. Residents appreciated the assistance with setting small goals that were tailored to them. The PPs supported residents to identify barriers and strategies to overcome them such as access to low cost or free programs, which was seen as an important step.

*“I actually learned a lot as well about how she [PP] handled the goal setting… she would say, “Okay, are there any challenges that would get in the way of you doing this?” And then I said, well, actually yes, you know, these three things would probably stop me. She said, “Now, let’s figure out how we get over those.” And I thought that was really important.”* (Post-visit FG5)

Shared goal setting with the PP was also important as residents felt involved in decision- making about their own health. PP tools were perceived as accessible, easy to use and provided good follow-up reminders for residents. The offer of home visits was considered an enabler of participation since residents had limited transportation options. Moreover, residents spoke positively about DRHD and public health nurses as trusted sources of health information. PPs were also viewed as knowledgeable about existing community resources and were able to link participants to them. Residents saw BETTER HEALTH as an asset for the community because it addressed a disease prevention gap.

### Feeling listened to and being understood were critically important when interacting with PPs about their health

In the post-visit phase, residents said it was important to feel heard and understood when engaging with a professional about their health issues.

*“I found she listened so well… Like before giving me advice, she took the time to listen to everything that I had to say. So I felt very understood.”* (Post-visit FG5)

Residents felt listened to by both the research assistant (RA) during baseline data collection and by the PP. The RA interview was identified by residents as the first step of building trust and rapport as it prompted reflection and inspired changes in behaviour. Residents also described the PP as having good listening skills, being professional and non-judgmental, and that they felt cared for, respected, and understood. The PP visit was described as private and comfortable and participants did not feel rushed.

## Discussion

In this study, we adapted the original BETTER intervention for a cRCT (BETTER HEALTH) directed toward community residents living in low income neighbourhoods and with a public health nurse as the PP. In both adaptation and the post-visit evaluation phases, we found that residents faced significant intersecting health and social challenges in coping with everyday life. A substantial contributor to stress was perceived to be living with poverty, coping with previous or current mental health issues or addictions, loneliness, and social isolation. Consequently, it was important that the adapted intervention incorporated resources for social and income support, food security support, and other resources (e.g., community social programs, community kitchens, mental health supports). PPs assisted participants to access these resources since many residents did not know how to access them.

We also found that disease prevention was not a priority for some community residents due to health and social challenges; they could only consider making lifestyle changes when their life was in a stable place. Similarly, Crooks et al. (2021) found that chronically ill residents from low income neighbourhoods reported only seeking medical care at walk-in clinics and emergency departments when they hit a “crisis point” rather than practicing disease prevention [29]. In our study, the supportive PP visits that incorporated health promotion and shared decision-making served as a “wake-up call” for many residents and helped them plan concrete strategies to improve their health.

Our research highlighted that feeling listened to was especially important when interacting with PPs. This finding supported the appropriateness of having public health nurses with strong skills in trust-building as PPs. Dupéré et al. (2012) reported that men living in deep poverty in Montreal were reluctant to seek needed medical care or social services; many had experienced significant abuse and victimization which led to difficulties expressing their feelings and trusting others [30]. Other researchers also reported that a lack of trust in other people was an important barrier for chronically ill patients with complex social needs to engage with health care services [31].

We found that residents valued accessible community programs and the BETTER HEALTH approach of using established community resources. PPs referred residents to *existing* community resources within the region and avoided duplication of services. In this context, the role of the PP is an educator and a navigator with extensive knowledge of relevant community resources.

The BETTER HEALTH intervention based in the community and delivered by public health nurses as PPs was positively perceived by residents. The PPs helped residents to make lifestyle changes by focusing on achievable short-term goals which contributed to the success of the intervention. The results of qualitative evaluation were consistent with the results of the cRCT which showed that residents in the intervention arm achieved more eligible actions compared to those in the waitlist arm [14].

Numerous community-based interventions have attempted to increase cancer and cardiovascular disease (CVD) screening and improve health outcomes [32–37]. Some interventions were targeted specifically toward those who might experience systemic barriers to accessing healthcare such as those living in rural areas [36]. Systematic reviews have found that multicomponent interventions which include one-on-one and/or group education sessions through various community settings (e.g. faith-based organizations, public health, community health centres), utilizing community health workers/volunteers or nurses have been successful in increasing screening rates for cancer and CVD with some studies also showing improvements in patient health outcomes [32–34]. Krantz et al. (2013) and Shlay et al. (2011) both successfully used community health workers to improve patient CVD-related outcomes (e.g., diet, weight and blood pressure) through one-on-one interventions in public health settings that consisted of motivational interviewing and goal-setting, patient navigation and referrals to medical /community resources [36, 37]. While both of these previous studies focused on CVD outcomes, BETTER and the BETTER HEALTH adaptation are unique in effectively providing an evidenced-based comprehensive approach to CDPS, including associated lifestyle factors [7, 14].

The results of our qualitative evaluation are consistent with a previous evaluation of the BETTER intervention that was conducted with patients in primary care living in urban, rural or remote communities in Newfoundland and Labrador [10, 11]. This previous evaluation reported that patients valued the PP visit which was perceived as personalized and comprehensive, the PPs were viewed as professional and had strong interpersonal skills, and patients were concerned about access to disease prevention [10]. Our study provides additional information about the perceived health of community residents living in low income neighbourhoods including stress and loneliness, the role of personal motivation, and the positive influence of the PP visit in helping residents achieve their personal health goals. The new PP role was largely consistent with the chronic disease prevention mandate of the public health department that participated in the study but was delivered in a one-to-one visit. The role of the PP public health nurse as an educator and a navigator allowed a more targeted approach focused on those most at need.

A particular strength of our study was the engagement of community stakeholders especially the community residents living in low income neighbourhoods who participated both as members of the CAC and in the adaptation phase of the study. The adapted intervention that was subsequently tested in the cRCT incorporated key features recommended by community residents such as having private and confidential visits with PPs who listened to concerns and helped residents to create personal goals that were meaningful. We speculate that this input from the community contributed to the positive results of the adapted intervention. The community residents also reinforced that the adapted intervention helped to fill a prevention gap in the community.

### Limitations

We acknowledge that many of the community residents who participated in focus groups or interviews may already have taken some steps to improve their health. These individuals may represent community members already empowered around health issues and who had the motivation to make lifestyle changes or to connect to community services. We do not know if individuals who felt unable to make lifestyle changes would have had the same positive views of the PP visit. Additionally, our study included community residents who volunteered to participate and we cannot be certain that the views of residents who did not participate would be similar. However, we recruited individuals from all eligible priority neighbourhoods in an effort to obtain a range of views and reached informational saturation of themes during the analysis [38]. In the post-visit evaluation, we enrolled about one-third of study participants who received the PP visit. We chose to include only those who had received a PP visit so we could obtain their impressions of the visit; however, it might have useful to have included residents who were eligible and consented but did not attend the visit. In doing so, we might have gained information about additional barriers that were unique to these individuals. In addition, only five men in the adaptation phase and four in the post-visit phase participated. As a result, we do not know whether we might have missed important information about the program. For example, we might have identified other opportunities to share information about BETTER HEALTH with men who might be otherwise reluctant to attend a PP visit. Another limitation is that we did not explore the cultural differences and approaches to behaviour change at the familial or community level beyond those identified by participants [39]. Therefore, our findings provide information about individual versus collective approaches to illness and health. A final limitation relates to the application of our intervention to a virtual setting. All PP visits were conducted in-person. Given that the onset of the Covid-19 pandemic and the switch to more virtual care, we are uncertain if our findings would be applicable in a virtual care setting.

## Conclusions

The adaptation phase was crucial to learn from community residents about their perceived health and to gauge acceptability of the BETTER HEALTH intervention. Significant challenges faced by community residents included those pertaining to mental health, loneliness and social isolation and living with poverty. Resources that addressed social needs were important additional components of the adapted intervention.

The post visit qualitative evaluation by community residents helped us understand key relational aspects of the PP visit including resident’s sense of being respected and understood. Residents perceived that help with setting personal and achievable goals empowered them to make changes. We also learned that the BETTER HEALTH intervention was viewed as providing access to chronic disease prevention in the community.

### Supplementary Information

**Supplementary Material 1. **

**Supplementary Material 2. **

## Data Availability

We are not able to publicly share the transcripts because we do not have consent from participants to do so. The analytic codes are available from the corresponding author upon reasonable request.

## Abbreviations

ADAPT-ITT Model: Assessment, Decision, Administration, Production, Topical Experts – Integration, Training, Testing
BETTER: Building on Existing Tools to Improve Chronic Disease Prevention and Screening in Family Practice
CAC: Community Advisory Committee
CBPR: Community-based participatory research
CDPS: Chronic disease prevention and screening
CHC: Community Health Centre
CMHA: Canadian Mental Health Association
DRHD: Durham Regional Health Department
PCEG: Primary Care Engagement Group
PP: Prevention practitioner
