# Supplementary material for: Adaptation and qualitative evaluation of the BETTER intervention for chronic disease prevention and screening by public health nurses in low income neighbourhoods: views of community residents
Source: BMC Health Serv Res. 2024 Apr 4;24:427. doi: 10.1186/s12913-024-10853-z (PMC10993474; doi:10.1186/s12913-024-10853-z)
Supplement: Supplementary file 1 — Supplementary Material 1. [file 12913_2024_10853_MOESM1_ESM.pdf]

Supplemental File 1: Consolidated criteria for reporting qualitative studies (COREQ): 32-item checklist

Domain 1: Research team and reflexivity

Personal Characteristics

1. Interviewer/facilitator Which author/s conducted the interview or focus group?

Response (R). Interviewers and focus groups were conducted by Mary Ann O'Brien (MAO), PhD and Tutsirai Makuwaza (TM) MA.

2. Credentials What were the researcher's credentials? E.g. PhD, MD

R. See Q1.

3. Occupation What was their occupation at the time of the study?

R. MAO is a researcher in the Department of Family and Community Medicine, University of Toronto. TM was a research associate in the same department at the time of the study.

4. Gender Was the researcher male or female?

R. MAO and TM are female.

5. Experience and training What experience or training did the researcher have?

R. MAO and TM have received training in qualitative research methods and have conducted qualitative research studies.

Relationship with participants

6. Relationship established Was a relationship established prior to study commencement?

R. Neither MAO or TM had a relationship with study participants prior to the study.

7. Participant knowledge of the interviewer

What did the participants know about the researcher? e.g. personal goals, reasons for doing the research.

R. During information sessions, potential participants met with TM and the purpose of the research was explained. At the beginning of each focus group or interview, MAO and TM introduced themselves and described their experience and personal motivations. The study purpose was provided in the consent forms.

8. Interviewer characteristics What characteristics were reported about the interviewer/facilitator? e.g. Bias, assumptions, reasons and interests in the research topic

R. MAO and TM were described as experienced qualitative researchers.

Domain 2: study design

Theoretical framework

9. Methodological orientation and Theory

What methodological orientation was stated to underpin the study? e.g. grounded theory,

discourse analysis, ethnography, phenomenology, content analysis

R. We used principles of community-participatory methods to guide the adaptation and the constant comparative method derived from grounded theory in the data analysis. We used the ADAPT-ITT model (Assessment, Decision, Administration, Production, Topical Experts – Integration, Training, Testing) for the adaptation.

Participant selection

10. Sampling How were participants selected? e.g. purposive, convenience, consecutive, snowball

R. Participants had to meet eligibility criteria (described in the paper).

11. Method of approach How were participants approached? e.g. face-to-face, telephone, mail, email

R. Participants were approached using multiple methods: printed flyers on bulletin boards at libraries and other community spaces, announcements on the Durham Regional Health Department Facebook page, displays at community events and in community agencies.

12. Sample size How many participants were in the study?

R. 38 community residents were included in the study.

13. Non-participation How many people refused to participate or dropped out? Reasons?

Three participants withdrew. One participant dropped out after a focus group because they decided it was not useful to them. Two potential participants declined to proceed with a focus group prior to its start because they did not wish to be identified on the consent form.

Setting

14. Setting of data collection Where was the data collected? e.g. home, clinic, workplace

R. Data were collected in residents homes or in community spaces such as library meeting rooms.

15. Presence of non-participants Was anyone else present besides the participants and researchers?

R. No one else was present during the focus groups and interviews.

16. Description of sample What are the important characteristics of the sample? e.g. demographic data, date

R. Adaptation: 14 community residents, 64% female; average age 54 y (range: 42 – 62 y). Post visit: 24 community residents, 83% female; average age 60 y (range: 43-63 y)

Data collection

17. Interview guide Were questions, prompts, guides provided by the authors? Was it pilot tested?

R. We created an interview guide that was pilot-tested.

18. Repeat interviews Were repeat interviews carried out? If yes, how many?

R. There were no repeat interviews

19. Audio/visual recording Did the research use audio or visual recording to collect the data?

R. We used audio recordings.

20. Field notes Were field notes made during and/or after the interview or focus group?

R. We created field notes during and after each focus group or interview.

21. Duration What was the duration of the interviews or focus group?

R. Each focus group was approximately one hour long. Interviews were about 45 min.

22. Data saturation Was data saturation discussed?

R. We reached data saturation and state this in the paper.

23. Transcripts returned Were transcripts returned to participants for comment and/or correction?

R. Transcripts were not returned to participants. Each transcript was checked for accuracy by TM.

Domain 3: analysis and findings

Data analysis

24. Number of data coders How many data coders coded the data?

R. There were 2 data coders.

25. Description of the coding tree Did authors provide a description of the coding tree?

R. We did not provide a description of the coding tree but it is available from the authors.

26. Derivation of themes Were themes identified in advance or derived from the data?

R. Themes were identified inductively from the data.

27. Software What software, if applicable, was used to manage the data?

R. NVivo was used for data management.

28. Participant checking Did participants provide feedback on the findings?

R. Participants did not provide feedback on the findings. We presented our findings several times to our Community Advisory Committee which included community residents.

Reporting

29. Quotations presented Were participant quotations presented to illustrate the themes / findings? Was each quotation identified? e.g. participant number

R. Supporting quotations were provided to illustrate the themes. Each quotation was identified using a number.

30. Data and findings consistent Was there consistency between the data presented and the findings?

R. We believed that there consistency between the data and the findings.

31. Clarity of major themes Were major themes clearly presented in the findings?

R. We believe that the major themes are clearly presented.

32. Clarity of minor themes Is there a description of diverse cases or discussion of minor themes?

We believe that the minor themes are clearly presented.

Reference:

Tong A, Sainsbury P, Craig J. Consolidated criteria for reporting qualitative research (COREQ): a 32-item checklist for interviews and focus groups. Int J Qual Health Care. 2007;19(6):349-357.
