# Supplementary material for: Adaptation and qualitative evaluation of the BETTER intervention for chronic disease prevention and screening by public health nurses in low income neighbourhoods: views of community residents
Source: BMC Health Serv Res. 2024 Apr 4;24:427. doi: 10.1186/s12913-024-10853-z (PMC10993474; doi:10.1186/s12913-024-10853-z)
Supplement: Supplementary file 2 — Supplementary Material 2. [file 12913_2024_10853_MOESM2_ESM.pdf]

Supplemental File 2

BETTER Health: Durham

Sample Interview and Focus Group Guides

1. Community Resident Adaptation Phase, Sample Interview and Focus Group Guides

2. Community Resident Post Prevention Practitioner Visit, Sample Interview and Focus Group Guides

BETTER Health: Durham

1. Community Resident Adaptation Phase

Sample Interview Guide

Preamble

[Introductions]

I would like to start by thanking you for taking the time to meet with me and to participate in this interview. For this interview, I am interested in your views and opinions on any steps you have taken or any care you have received to improve your general health and prevent chronic diseases such as diabetes or high sugars, high blood pressure or cancer. I will also talk to you about a proposed research program called BETTER Health: Durham. In this proposed research program, people may be eligible to receive a visit by a Prevention Practitioner nurse to help them take steps to prevent chronic diseases. During our discussion today, it is important for you to know that there are no right or wrong answers.

Your participation is entirely voluntary. You may stop the interview at any time or refuse to answer any questions. We would like to audio record this session if it is OK with you. Your answers will be kept confidential. This means that you will not be identified by your name and anonymous responses will only be shared amongst the research team. Any information we include in our report will not be associated with your name. Are you OK with being audio recorded? [*Proceed with taking notes if necessary*] I would also like to ask you to switch off your cell phone or change to silent mode.

[Begin Interview]

Opening question:

1. What do you think about the health of people in this neighbourhood?
   Probes [Note: specific probe used depended on response to the question]: Are people in general healthier than other neighbourhoods?
   Are people in general less healthy than in other neighbourhoods?
   What are some healthy behaviours that you see?
   What are some unhealthy behaviours that you see?

2. In your view, what leads some people to be healthy, and other people to be unhealthy? Let’s start with being healthy. What leads some people to be healthy?
   Probe: What about being unhealthy?

3. What are ways that people can improve their health, in general?

4. What can the people around you that is, in this neighbourhood do to improve their health?
   Probes: What helps people around you with doing these things?
   What are barriers or obstacles they face?

5. In the past 12 months, did **you** personally do anything to improve your health?
   Probes: For example, lost weight, quit smoking, increased exercise?
   How did that go for you?
   What worked, and what didn't?

6. What things affected your ability to do these activities [mention activities from #5]?
   Probes: What made it easier to do these activities?
   What made it harder to do these activities?
   Is there anything about this neighbourhood that made it easier to do these activities? [probe for neighbourhood characteristics]
   Is there anything about this neighbourhood that made it harder to do these activities? [probe for neighbourhood characteristics]

7. Are you worried about your risk of developing chronic diseases such as diabetes or high sugars, high blood pressure or cancer? Only share what you feel comfortable to share.
   Probes: If yes, please tell me why you think you are at risk.
   What type of disease do you think you could be at risk for?
   If no, please tell me why you think you are not at risk.

I would like to turn now to any care you have received to prevent chronic diseases such as diabetes or high sugars, high blood pressure or cancer.

8. Please tell me about any care you have received to prevent chronic diseases. Again only share what is comfortable to share.
   Probes: Where did you receive care?
   What type of care did you receive?
   [If they received care]: How did that go?

9. Did you have any trouble getting care to prevent chronic diseases?
   Probes: Do you currently have a family doctor or nurse practitioner?
   If yes, does your family doctor or nurse practitioner talk to you about preventing chronic diseases? If yes, how?

   If no to having a family doctor/nurse practitioner, have you experienced difficulties finding a family doctor or nurse practitioner?
   Probe: If yes, please tell me about what kinds of difficulties you've experienced.

10. Are there other difficulties with getting care to prevent chronic diseases?

    Probes: If yes, do you have any difficulty getting to your family doctor or nurse practitioner? Please tell me about what kinds of difficulties you've experienced. [probe on issues mentioned by participants. These may include as mobility, transportation, opening hours, getting to appointments]
    What about challenges taking time off from work?
    What about child care or caregiving?
    What about personal or family problems?

Turning now to the proposed research program called BETTER Health: Durham

[Explain proposed research program]

In this proposed research program, a specially trained nurse, working out of a public health department will be available to meet with people to help them to improve their health by receiving tests and activities that can prevent chronic diseases such as diabetes or high sugars, high blood pressure or cancer.

11. What do you think about someone meeting with a nurse to improve their health?
    Probes: What do you like about meeting with a nurse?
    What don't you like about meeting with a nurse? Would you rather meet with another health professional such as a family doctor?

12. What do you think about having a nurse available to meet with people from this neighbourhood to improve their health?
    Probes: What do you like about meeting with a nurse?
    What don't you like about meeting with a nurse? Would you rather meet with another health professional such as a family doctor?

13. What would make it easier for people from this neighbourhood to meet with a nurse?

14. What would make it harder for people from this neighbourhood to meet with a nurse?

15. For some people, the nurse might suggest having tests such as a mammogram, pap smear or colorectal cancer screening. For these tests, more would need to be done.
    Probe: What are the barriers to doing these tests?
    What would make it easier to do these tests? (e.g. mammogram clinics held in the neighbourhood)

16. In your view, how could we reach people in your neighbourhood to tell them about the proposed research program?
    Probes: Are there specific people or organizations we should work with?

What is the best way to reach them?
Are there really good places to put up posters, so people see them?
What about neighbourhood newsletters, email lists, FaceBook pages or other means?

Is there anything else you would like to tell me?

Thank you very much for your time. I really appreciate it.

BETTER Health: Durham

Community Resident Adaptation Phase

Sample Focus Group Guide

Preamble

[Introductions]

I would like to start by thanking you for taking the time to meet with me and to participate in this focus group. For our discussion today, I am interested in your views and opinions on any steps you have taken or any care you have received to improve your general health and prevent chronic diseases such as diabetes or high sugars, high blood pressure or cancer. I will also talk to you about a proposed research program called BETTER Health: Durham. In this proposed research program, people may be eligible to receive a visit by a Prevention Practitioner nurse to help them take steps to prevent chronic diseases. During our discussion today, it is important for you to know that there are no right or wrong answers.

Your participation is entirely voluntary. You may leave the focus group at any time or refuse to answer any questions. We will be audiorecording this session today. As a reminder, your answers will be kept confidential. This means that you will not be identified by your name and anonymous responses will only be shared amongst the research team. Any information we include in our report will not be associated with your name.

Just a few housekeeping things. The session will go approximately an hour and a half. Since we will not take any breaks, please help yourself to snack and drinks during the session. We would also like to ask you to switch off your cell phones or change to silent mode.

[Begin Focus group]

Opening question:

1. What do you think about the health of people in this neighbourhood?
   Probes [Note: specific probe depended on response to the question]: Are people in general healthier than other neighbourhoods?
   Are people in general less healthy than in other neighbourhoods?
   What are some healthy behaviours that you see?
   What are some unhealthy behaviours that you see?

2. In your view, what leads some people to be healthy, and other people to be unhealthy? Let’s start with being healthy. What leads to some people to be healthy?
   Probe: What about being unhealthy?

3. What are ways that people can improve their health, in general?

4. What can the people around you that is, in this neighbourhood do to improve their health?
   Probes: What helps people around you with doing these things?
   What are barriers or obstacles they face?

5. In the past 12 months, did **you** personally do anything to improve your health?
   Probes: For example, lost weight, quit smoking, increased exercise?
   How did that go for you?
   What worked, and what didn't?

6. What things affected your ability to do these activities [mention activities from #5]?
   Probes: What made it easier to do these activities?
   What made it harder to do these activities?
   Is there anything about this neighbourhood that made it easier to do these activities? [probe for neighbourhood characteristics]
   Is there anything about this neighbourhood that made it harder to do these activities? [probe for neighbourhood characteristics]

7. Are you worried about your risk of developing chronic diseases such as diabetes or high sugars, high blood pressure or cancer? Only share what you feel comfortable to share.
   Probes: If yes, please tell me why you think you are at risk.
   What type of disease do you think you could be at risk for?
   If no, please tell me why you think you are not at risk.

I would like to turn now to care you may have received to prevent chronic diseases such as diabetes or high sugars, high blood pressure or cancer.

8. Please tell me about any care you have received to prevent chronic diseases. Again only share what is comfortable to share.
   Probes: Where did you receive care?
   What type of care did you receive?
   [If they received care]: How did that go?

9. Have you had any trouble getting care to prevent chronic diseases?
   Probes: Do you currently have a family doctor or nurse practitioner?
   If yes, does your family doctor or nurse practitioner talk to you about preventing chronic diseases? If yes, how?

   If no to having a family doctor/nurse practitioner, have you experienced difficulties finding a family doctor or nurse practitioner?
   Probe: If yes, please tell me about what kinds of difficulties you've experienced.

10. Are there other difficulties with getting care to prevent chronic diseases?

    Probes: If yes, do you have any difficulty getting to your family doctor or nurse practitioner? Please tell me about what kinds of difficulties you've experienced. [probe on issues mentioned by participants. These may include as mobility, transportation, opening hours, getting to appointments]
    What about challenges taking time off from work?
    What about child care or caregiving?
    What about personal or family problems?

Turning now to the proposed research program called BETTER Health: Durham
[Explain proposed research program]

In this proposed research program, a specially trained nurse, working out of a public health department will be available to meet with people to help them to improve their health by receiving tests and activities that can prevent chronic diseases such as diabetes or high sugars, high blood pressure or cancer.

11. What do you think about someone meeting with a nurse to improve their health?
    Probes: What do you like about meeting with a nurse?
    What don't you like about meeting with a nurse? Would you rather meet with another health professional such as a family doctor?

12. What do you think about having a nurse available to meet with people from this neighbourhood to improve their health?
    Probes: What do you like about meeting with a nurse?
    What don't you like about meeting with a nurse? Would you rather meet with another health professional such as a family doctor?

13. What would make it easier for people from this neighbourhood to meet with a nurse?

14. What would make it harder for people from this neighbourhood to meet with a nurse?

15. For some people, the nurse might suggest having tests such as a mammogram, pap smear or colorectal cancer screening. For these tests, more would need to be done.
    Probe: What are barriers to doing these tests?
    What would make it easier to do these tests? (e.g. mammogram clinics held in the neighbourhood)

16. In your view, how could we reach people in your neighbourhood to tell them about the proposed research program?
    Probes: Are there specific people or organizations we should work with?
    What is the best way to reach them?
    Are there really good places to put up posters, so people see them?

What about neighbourhood newsletters, email lists, FaceBook pages or other means?

Is there anything else you would like to tell me?

Thank you very much for your time. I really appreciate it.

BETTER Health: Durham

2. Community Resident Post Prevention Practitioner Visit

Sample Interview and Focus Group Guides

BETTER Health: Durham

Community Resident Post Prevention Practitioner Visit

Sample Interview Guide

Preamble

[Introductions]

I would like to start by thanking you for taking the time to meet with me and to participate in this interview. For this interview, I am interested in your views and opinions on any steps you have taken or any care you have received to improve your general health and prevent chronic diseases such as diabetes or high sugars, high blood pressure or cancer. I will also talk to you about the research program called BETTER Health: Durham. In this research program, people like you received a visit from a Prevention Practitioner public health nurse to help them take steps to prevent chronic diseases. During our discussion today, it is important for you to know that there are no right or wrong answers.

Your participation is entirely voluntary. You may stop the interview at any time or refuse to answer any questions. We would like to audio record this session if it is OK with you. Your answers will be kept confidential. This means that you will not be identified by your name and anonymous responses will only be shared amongst the research team. Any information we include in our report will not be associated with your name. Are you OK with being audio recorded? [*Proceed with taking notes if necessary*] I would also like to ask you to switch off your cell phone or change to silent mode.

[Begin Interview]

Opening question:

1. What do you think about the health of people in your neighbourhood?
   Probes [Note specific probe depended on response to question]: Are people in general healthier than other neighbourhoods?
   Are people in general less healthy than in other neighbourhoods?
   What are some healthy behaviours that you see?
   What are some unhealthy behaviours that you see?

2. In your view, what leads some people to be healthy, and other people to be unhealthy? Let's start with being healthy. What leads some people to be healthy?
   Probe: What about being unhealthy? Are there issues that people are struggling with such as social isolation? What about mental health issues like depression?

3. What are ways that people can improve their health, in general?

4. What can the people around you that is, in your neighbourhood do to improve their health?
   Probes: What helps people around you with doing these things?
   What are barriers or obstacles they face?

5. Are you worried about your risk of developing chronic diseases such as diabetes or high sugars, high blood pressure or cancer? Only share what you feel comfortable to share.
   Probes: If yes, please tell me why you think you are at risk.
   What type of disease do you think you could be at risk for?
   If no, please tell me why you think you are not at risk.

Before your meeting with the nurse, you had a meeting with the research assistant [name].

6. What did you think about the meeting with the [name, research assistant]?
   Probes: What did you think about the questions she asked?

7. Did you begin any new activities to improve your health after you met with her?

I would like to turn now to the visit you had with the Prevention Practitioner public health nurse.

8. Did you have any difficulties setting up a meeting with the nurse?
   Probes: Please tell me about the kinds of difficulties you've experienced. [probe on issues mentioned by participants. These may include as mobility, transportation, getting an appointment]
   What about challenges taking time off from work?
   What about child care or caregiving?
   What about personal or family problems?

9. What things made it easier for you to set up the meeting with the nurse?
   Probes: What about the location of the meeting?

10. What do you think about your visit with the nurse?
    Probes: What did you like about the meeting with the nurse?
    What didn't you like about the meeting with the nurse?

11. During the meeting, the nurse used several tools. What did you like about these tools? What didn't you like about the tools? [if people don't remember, show examples of the tools] [Interviewer: Review each tool separately]

12. Since your meeting with the nurse, were you able to work on any of your goals? Remember there are no right or wrong answers. How did that go for you?
    Probes: What made it harder or easier to work on your goals?

13. For some people, the nurse suggested having tests such as a mammogram, pap smear or colorectal cancer screening. For these tests, more had to be done. In your situation, did the nurse suggest that you have any tests? Only share what is comfortable to share.
    Probes: [If yes] Were you able to complete these tests?
    [If yes], what made it easier to complete these tests.
    [if no], what made it hard to complete these tests?

Thank you for sharing your experiences.

I would like to turn now to activities you may already have been doing before the nurse visit, to prevent chronic diseases such as diabetes or high sugars, high blood pressure or cancer.

14. In the past 12 months, did **you** personally do anything to improve your health prior to the nurse visit?
    Probes: For example, lost weight, quit smoking, increased exercise?
    How did that go for you?
    What worked, and what didn't?
    When did you begin these activities?

15. What made it easier to do these activities [mention activities from #14]?
    Probes: What made it harder to do these activities?

I would now like to ask more general questions about the study.

16. How did you first hear about the study?

17. What kinds of things helped you to decide to participate in the study?

18. Do you think both men and women you know would be interested in participating in a program like BETTER HEALTH: Durham?
    Probes: Are there different ways of reaching either men or women to participate in a program like BETTER HEALTH: Durham?

19. What is the single most important barrier to continuing a program like BETTER Health: Durham?

20. What is the single most important thing that would make it easier to continue a program like BETTER Health: Durham?

21. In the future, how could we reach people in your neighbourhood to tell them about a program like BETTER Health: Durham?
    Probes: Are there specific people or organizations we should work with?
    What is the best way to reach them?

Are there really good places to put up posters, so people see them? What about neighbourhood newsletters, email lists, FaceBook pages or other means?

Is there anything else you would like to tell me?

Thank you very much for your time. I really appreciate it.

BETTER Health: Durham

Community Resident Post Prevention Practitioner Visit

Sample Focus Group Guide

Preamble

[Introductions]

I would like to start by thanking you for taking the time to meet with me and to participate in this focus group. For our discussion today, I am interested in your views and opinions on any steps you have taken or any care you have received to improve your general health and prevent chronic diseases such as diabetes or high sugars, high blood pressure or cancer. I will also talk to you about the research program called BETTER Health: Durham. In this research program, people were eligible to receive a visit by a Prevention Practitioner nurse to help them take steps to prevent chronic diseases. During our discussion today, it is important for you to know that there are no right or wrong answers.

Your participation is entirely voluntary. You may leave the focus group at any time or refuse to answer any questions. We will be audiorecording this session today. As a reminder, your answers will be kept confidential. This means that you will not be identified by your name and anonymous responses will only be shared amongst the research team. Any information we include in our report will not be associated with your name.

Just a few housekeeping things. The session will go approximately an hour and a half. Since we will not take any breaks, please help yourself to snack and drinks during the session. We would also like to ask you to switch off your cell phones or change to silent mode.

[Begin Focus group]

Opening question:

1. What do you think about the health of people in this neighbourhood?
    Probes [Note specific probe depended on response to question]: Are people in general healthier than in other neighbourhoods?
    Are people in general less healthy than other neighbourhoods?
    Are there issues that people are struggling with such as social isolation, mental health issues such as depression?
    What are some healthy behaviours that you see?
    What are some unhealthy behaviours that you see?

2. In your view, what leads some people to be healthy, and other people to be unhealthy? Let's start with being healthy. What leads some people to be healthy?
    Probe: What about being unhealthy?

3. What are ways that people can improve their health, in general?

4. What can the people around you that is, in this neighbourhood do to improve their health?
   Probes: What helps people around you with doing these things?
   What are barriers or obstacles they face?

5. Are you worried about your risk of developing chronic diseases such as diabetes or high sugars, high blood pressure or cancer? Only share what you feel comfortable to share.
   Probes: If yes, please tell me why you think you are at risk.
   What type of disease do you think you could be at risk for?
   If no, please tell me why you think you are not at risk.

Before your meeting with the nurse, you had a meeting with the research assistant [name].

6. What did you think about the meeting with [name, research assistant]?
   Probes: What did you think about the questions she asked from the questionnaire?

7. Did you begin any new activities to improve your health after you met with her?

I would like to turn now to the visit you had with the Prevention Practitioner public health nurse.

8. Did you have any difficulties setting up a meeting with the nurse?
   Probes: Please tell me about the kinds of difficulties you've experienced. [probe on issues mentioned by participants. These may include as mobility, transportation, getting an appointment]
   What about challenges taking time off from work?
   What about child care or caregiving?
   What about personal or family problems?

9. What things made it easier for you to set up the meeting with the nurse?
   Probes: What about the location of the meeting?

10. What do you think about your visit with the nurse?
    Probes: What did you like about the meeting with the nurse?
    What didn't you like about the meeting with the nurse?

11. During the meeting, the nurse used several tools. What did you like about these tools? What didn't you like about the tools? [if people don't remember, show examples of the tools] [Interviewer: Review each tool separately]

12. Since your meeting with the nurse, were you able to work on any of your goals? Remember there are no right or wrong answers. How did that go for you?
    Probes: What made it harder or easier to work on your goals?

13. For some people, the nurse suggested having tests such as a mammogram, pap smear or colorectal cancer screening. For these tests, more had to be done. In your situation, did the nurse suggest that you have any tests? Only share what is comfortable to share.
    Probes: [If yes] Were you able to complete these tests?
    [If yes], what made it easier to complete these tests.
    [if no], what made it hard to complete these tests?

Thank you for sharing your experiences with the nurse.

I would like to turn now to activities you may already have been doing before the nurse visit to prevent chronic diseases such as diabetes or high sugars, high blood pressure or cancer.

14. In the past 12 months, did **you** personally do anything to improve your health?
    Probes: For example, lost weight, quit smoking, increased exercise?
    How did that go for you?
    What worked, and what didn't?

15. What things affected your ability to do these activities [mention activities from #14]?
    Probes: What made it easier to do these activities?
    What made it harder to do these activities?
    Is there anything about this neighbourhood that made it easier to do these activities? [probe for neighbourhood characteristics]
    Is there anything about this neighbourhood that made it harder to do these activities? [probe for neighbourhood characteristics]

Thank you for sharing your experiences with the nurse. I would now like to ask more general questions about the study.

16. How did you first hear about the study?

17. What kinds of things helped you to decide to participate in the study?

18. Do you think both men and women would be interested in participating in a program like BETTER HEALTH: Durham?
    Probes: Are there different recruitment strategies needed for either men or women?

19. What is the single most important barrier to continuing a program like BETTER Health: Durham?

20. What is the single most important thing that would make it easier to continue a program like BETTER Health: Durham?

21. In the future, how could we reach people in your neighbourhood to tell them about a program like BETTER Health: Durham?
    Probes: Are there specific people or organizations we should work with?
    What is the best way to reach them?
    Are there really good places to put up posters, so people see them?
    What about neighbourhood newsletters, email lists, FaceBook pages or other means?

Is there anything else you would like to tell me?

Thank you very much for your time. I really appreciate it.
